# Efficacy of oral meloxicam suspension for prevention of pain and inflammation following band and surgical castration in calves

**DOI:** 10.1186/s12917-016-0735-3

**Published:** 2016-06-13

**Authors:** M. E. Olson, Brenda Ralston, Les Burwash, Heather Matheson-Bird, Nick D. Allan

**Affiliations:** Alberta Veterinary Laboratories, 411 19th Street SE, Calgary, Alberta T2E 6J7 Canada; Alberta Agriculture and Forestry, 97 East Lake Ramp NE, Airdrie, Alberta, T4A 0C3 Canada; Alberta Horse Industry Association, 97 East Lake Ramp NE, Airdrie, Alberta, T4A 0C3 Canada; Chinook Contract Research, 97 East Lake Ramp NE, Airdrie, Alberta, T4A 0C3 Canada

**Keywords:** Oral meloxicam, Castration, Behavior, Pain, Calves

## Abstract

**Background:**

Castration is one of the most common procedures performed on beef and dairy cattle. The objective of the study was to determine the efficacy of meloxicam oral suspension in reducing pain and inflammation in calves following band or surgical castration.

**Methods:**

Two identical trials with the exception of the method of castration (Band Castration Study 1 and Surgical Castration Study 2) were conducted. Sixty (60) healthy Holstein calves 4 to 5 months of age (138–202 Kg) were used. Animals received either Meloxicam Oral Suspension at a dose of 1 mg/kg BW (*n =* 15 Study 1 and 15 Study 2) or Saline (*n =* 15 Study 1 and 15 Study 2) 2 h before castration. Physiological (Heart Rate, Plasma Cortisol and Plasma Substance P) and Behavioral (Visual Analog Scale (VAS), Accelerometers and tail Pedometers) evaluations were conducted before (day -1) and after Castration (Day 0, 1, 2, 3). Inflammation was evaluated daily by providing an individual animal score (Study1) or with a measurement of scrotal thickness (Study 2).

**Results:**

Heart rates were significantly greater in control animals following band and surgical castration. Plasma cortisol and substance P were significantly reduced in animals receiving Meloxicam Oral Suspension. Control animals had significantly greater VAS scores. Accelerometers showed that meloxicam treated animals had a significantly greater motion index and number of steps as well as less % time lying and number of lying bouts. The scrotal inflammation (based on scrotal swelling) was significantly decreased in the meloxicam treated animals compared to the control animals on day 1, day 2 and 3.

**Conclusion:**

Meloxicam Oral Suspension was able to significantly reduce the display of painful behaviors and physiological responses to pain in band castrated and surgical castrated calves for up to 72 h following a single oral treatment of 1 mg/kg body weight. Meloxicam Oral Suspension was able to significantly reduce scrotal inflammation in band castrated and surgical castrated calves.

## Background

Castration is one of the most common procedures performed on male beef and dairy cattle. The benefits of castration are well established and include population control, reduced aggressive and mounting behaviors and improved carcass quality. Regardless of the method, castration has been shown to produce physiological, behavioral and neuroendocrine alteration associated with pain and distress [1–5]. Currently, approved North American local and general anesthetics available to veterinarians and producers to control pain and stress associated with castration effects are only short term [3]. Recently, oral meloxicam has been demonstrated to provide effective pain relief in calves following dehorning and castration [6, 7] with efficacy duration of up to 3 days and a plasma half-life of 27 h [8]. Meloxicam is a nonsteroidal anti-inflammatory drug (NSAID) of the oxicam class which acts by inhibition of prostaglandin synthesis, thereby exerting anti-inflammatory, analgesic and anti-pyretic properties [8, 9]. Meloxicam preferentially inhibits the COX-2 isoenzyme and has been shown to be safer than non-selective NSAIDs like aspirin, flunixin and ketoprofen [10]. Meloxicam Oral Suspension is a new product that has been developed and recently registered in Canada. Meloxicam Oral Suspension has advantages over injectable formulations: 1) The onset of therapeutic activity is similar between injectable and oral formulations but the oral formulation has a significantly longer duration of activity (8), 2) Injection site reactions and needle stick injuries can be avoided with the use of an oral formulation, 3) Meloxicam Oral Suspension is highly palatable and is readily taken by oral gavage and it can be top dressed onto feed as a delivery method. 4) Meloxicam Oral Suspension is suitable for use in a number of domestic and wildlife mammalian species where restraint required for injection is not possible. The objective of the study was to determine the efficacy of meloxicam oral suspension in reducing pain and inflammation in calves following band or surgical castration. This study contributed to the registration of Meloxicam Oral Suspension in Canada for “alleviation of pain and inflammation following surgical and band castration in cattle”.

## Methods

The study was conducted in compliance with the guidelines of the Canadian Council on Animal Care after the appropriate review by the Institutional Animal Care and Use Committee. In evaluation of the study consideration was given that no local anesthetic was provided to animals and although not advocated it is standard of practice in North America. The early behavioral and physiological effects (first 3 h) of meloxicam oral suspension could not be evaluated if local anesthesia was employed. A pre-study power calculation was performed to determine the number of animals required to generate meaningful results. Procedures were designed to avoid or minimize discomfort, distress and pain to the animals.

Two identical trials, with the exception of the method of castration, were conducted during the month of August, 2014. The trails were run at separate times and there was no overlap of trials. Animals received Meloxicam Oral Suspension at an oral dose of 1 mg/kg BW (*n =* 15 Study 1 and *n =* 15 Study 2) or oral Saline 1 mL per 15 kg (*n =* 15 Study 1 and *n =* 15 Study 2) approximately 2 h before castration. The person in charge of the preparation of dosing syringes, treating the animals and randomization was not blinded. The persons caring for animals, collecting blood, downloading heart rate and accelerometer data, behavior scoring the animals, evaluating inflammation and analyzing the plasma were blinded.

Holstein calves were 4 to 5 months of age 138–186 Kg (Study 1, *n =* 15 for treatment, *n =* 15 for control); 142–202 Kg (Study 2, *n =* 15 for treatment, *n =* 15 for control). For each study animals were weighed, ranked by weight and allocated to treatment or control using random numbers. The animal was the experimental unit. All calves were brought to the facility from multiple sources in Southern Alberta, Canada at less than 4 weeks of age, fed a milk diet for 2–4 weeks and placed on solid feed. The feed at the time of study was a mixed barley grain and alfalfa hay ration (77.4 % dry matter, 12 % crude protein) with a mineral/vitamin supplement. Before and during the study, animals were comingled in a rectangular pen (24 m x 30 m) with 10 m of concrete feed bunk, automatic waterer and shelter (5 m x 10 m). Calves had free choice feed and were provided fresh feed twice daily. One animal was removed from the Meloxicam surgical castrated group due to a surgical complication which resulted in excess bleeding and the need for further care.

### Castration procedure

In North America it is not standard practice to provide local or general anesthesia at the time of castration. All castrations were performed at time 0 (approximately two hours after receiving the treatment with Meloxicam Oral Suspension (Alberta Veterinary Laboratories, Calgary, AB) or saline (Baxter, Mississauga, ON, Canada). Band castrations were performed, without local anesthesia, by placing a latex band (UFA Coop, Calgary, Alberta) around the neck of the scrotum, employing a banding tool (UFA Coop, Calgary, Alberta)). Surgical castrations were performed without local anesthetics. The scrotum was disinfected with a chlorhexidine surgical scrub (Hibitane Skin Cleanser, Zoetis, Kirkland, Quebec, Canada). Longitudinal incisions were made on the lateral sides of the scrotum. The testes and spermatic cord were exteriorized by blunt dissection and the cremastor was broken using manual traction. Each testis was extracted with a slow steady pull.

### Heart rate

The hair over the ventral girth was clipped. Girth heart rate recorders and Heart Rate Data loggers (WM Smartsync Heart Rate Logger, Oregon Scientific, PO box 1190, Cannon Beach Oregon) were placed around the girth of each animal on day 0 at the time of treatment (approximately -2 h). They were held in place with adhesive tape (3” Adhesive Ultra Elastic Tape, Covidien Canada, Saint Laurent, Quebec) wrapped loosely around the chest. Data loggers and straps were removed and data was downloaded on day 1, approximately 24 h after placement. Heart rates were continually collected with each data point representing the heart rate over a 10 s period. Heart Rates were downloading using a laptop computer and software supplied by the manufacturer (WM Smartsync Heart Rate Logger, Oregon Scientific, PO box 1190, Cannon Beach Oregon).

### Blood collection and cortisol and substance P analysis

Blood (10 mL) was collected by jugular or tail vein venipuncture on day -1, t = 5 h, t = 24 h, t = 48 h and t = 72 h. Ten (10) mL of blood was collected for Cortisol and Substance P (SP) analysis. Blood was collected in heparinized tubes and aprotonin (0.1 mL) was immediately added. Blood samples were centrifuged at 1600 x g for 15 min in a refrigerated centrifuge. The plasma was separated and placed in microfuge tubes in a -80C freezer for analysis. Cortisol and Substance P were analyzed using validated assay kits (Assay Designs™ Cortisol enzyme immunoassay (EIA) kit, Assay Designs, Inc. Ann Arbor, MI, USA;

Parameter, Substance P Assay, R&D Systems, Inc.614 McKinley Place NE, Minneapolis, MN USA).

### Accelerometer recording

Accelerometers (IceTags, www.icerobotics.com) were used to objectively measure movements of the animals. IceTags are a specialized activity monitoring system developed by IceRobotics Ltd, Edinburgh, Scotland, UK to support research into livestock behaviour, health and welfare. They have been validated for cattle, sheep and goats (www.icerobotics.com, [11]). The system consists of an IceTag Sensor which is a 3 axis accelerometer that is strapped to the hind leg of an animal and a download station. Data was collected continually over the study period. The sensor outputs include: 1) Standing/Lying: determined by the sensor passing a specific threshold between horizontal/vertical, 2) Lying bouts: exact start and end time of each lying bout, plus duration, 3) Motion index: indicates the overall activity of the animal calculated using the acceleration on each of the 3 axes. This is a manufacturer proprietary measure and is recommended over the step count as a measure of activity and 4) Step count: the number of times the calf lifts their tagged leg, based on the amount of force the animal uses.

ICE Tag recorders were placed on the left hind hock of each animal on day -1. The ICE tags were secured with tape (3” Adhesive Ultra Elastic Tape, Covidien Canada, Saint Laurent, Quebec). The time clock on the recorders was synchronized and set to the current date and time of the download station. ICE tags remained in place until day 3 (approximately 96 h after the treatment with meloxicam or saline placebo). The information was downloaded for analysis. Downloading was performed in the laboratory after using a reader, laptop computer and software supplied by the manufacturer IceRobotics (IceRobotics Ltd, Edinburgh, Scotland, UK).

After the data was downloaded the following data was excluded for analysis: 1.) The data from time of activating the ICE Tag until the device was placed on the animal’s leg and the animal returned to the home pen. 2.) The data during the period of time animals were being processed (Blood collection, castration, tail pedometer placement and removal, heart rate monitor placement and removal, movement of animals to and from handling area) and 3.) The data from the time of ICE tag removal until downloading of the data. The sum of each activity (standing time, lying time, lying bouts, motion index and step counts) were tabulated for each study period.

### Visual behavioral monitoring of pain

Behavioral data were obtained as described below. All samples were taken at the times relative to when (day and hour) a particular group was castrated. To assess treatment effects, behavioral observations were made by three experienced observers that were blind to the treatments. Observers were experienced animal scientists with training from an ethologist (Dr. Schwartzkopf-Gerswin) well recognized in recording painful behaviors. Observers used a Visual Analog Scale (VAS) to document behavioral responses indicative of pain and discomfort: Day 0: One (1) hour after castration for approximately 3 h (there were more painful behaviors so recording took more time); Day 1: Two hours of observation; Day 2: Two hours of observation; Day 3: Two hours of observation. The scoring was based upon the presence or absence of the following behaviors :belly kicking, stretching, changing of position, arching of the back, standing alone in pen, looking or attempting to lick the scrotum, frequent tail flicking, not alert and lack of interest in feed or water. The VAS was a 100 mm horizontal line with the far left indicating no pain response and the far right representing an extreme pain response. The observers placed a mark along this continuum that represented the amount of pain response an animal was exhibiting. The distance from the end point to the mark was measured to the nearest 0.1 cm and was recorded as the animal’s response to the castration (VAS score).

### Tail movement (tail pedometers)

Pedometers (Waterproof Step Movement Calorie Counter Multi-Function Digital Pedometer M2, China) were used to determine tail movements. Pedometers were secured to the tail of each animal (10 cm from the tail base) on day -1 with adhesive tape (2” Adhesive Ultra Elastic Tape, Covidien Canada, Saint Laurent, Quebec). Pedometer values were recorded and reset at approximate times t = 0, t = 24 h, t = 48 h and t = 72 h. The pedometers were manually read and the “steps” recorded. After recording, the pedometers were reset and placed on the tail for the data collection for the next day. Pedometers were removed on day 3.

### Inflammation evaluation

In band castrated animals the scrotal tissue swelling above the band was scored post castration on day 0 (immediately after castration), day 1, day 2 and day 3 at the time of blood collection (when animal was restrained in head gate). These scores were recorded as: 0 = No swelling, 1 = slight swelling, 2 = moderate swelling and 3 = severe swelling. The diameter of the mid scrotum tissue was measured post castration in surgically castrated animals on day 0 (immediately after castration), day 1, day 2 and day 3 at the time of blood collection (when animal was restrained in head gate). This was performed using digital calipers.

### Data analysis

A two-tailed Student’s *t* test was used to compare treatment and control heart rates, cortisol concentrations, substance P concentrations, accelerometer data, pedometer data and scrotal thickness measurements. Non-parametric analysis (Mann–Whitney test) was used to compare scrotal thickness scores and visual analog scale values. Significance was established at a 95 % confidence interval and data is expressed as mean, standard error (SE) with *P* values.

## Results

### Heart rate

The mean heart rates were recorded between the following times: 1) 2 to 4 h after castration, 2) 6 to 8 h after castration and 3) 8 to 10 h after castration. The mean heart rate was calculated using the software provided by the Data Logger provider (WM Smartsync Heart Rate Logger, Oregon Scientific, PO box 1190, Cannon Beach Oregon). During these times animals were not being processed and the recordings were free of interference. In many of the animals signals could not be obtained after 10 h of recording so data was not analyzed past this time. The data is summarized in Table 1. The heart rates were significantly elevated in the control animals compared to the treated animals (*P <* 0.05) for both the band castrated and surgical castrated animals for all observation times.

**Table 1:** Heart Rates of Calves following Band or Surgical Castration

		Band Castration			Surgical Castration		
Time Post – Castration	Treatment	Mean (beats/min)	SE	*P* Value	Mean (beats/min)	SE	*P* Value
2–4 h	Meloxicam	119.3	2.2	0.0001	100.2	4.0	0.0019
	Control (Saline)	141.2	2.7		116.6	2.0	
6–8 h	Meloxicam	112.9	2.0	0.0005	98.4	5.1	0.0003
	Control (Saline)	131.3	3.4		122.9	3.0	
8–10 h	Meloxicam	113.3	3.9	0.0033	103.2	3.6	0.0026
	Control (Saline)	134.8	4.6		119.0	2.7	

### Plasma cortisol and substance P

**Fig. 1:**
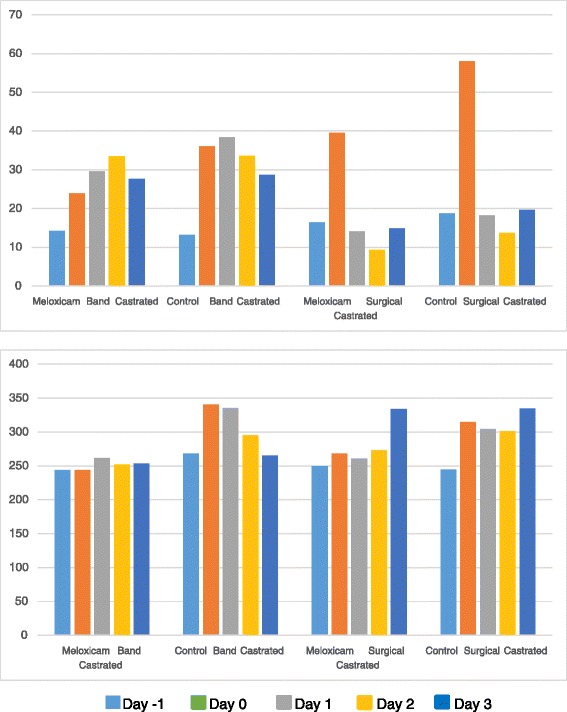
Plasma Cortisol (Upper, ηmol/L) and Plasma Substance P (Lower, pg/L) in band and surgically castrated calves on day -1, 0, 1, 2 and 3

Animals were acclimated to be handled and minimal to no restraint was required to collect blood from the tail vein of calves. Therefore the act of blood collection had minimal effects on plasma cortisol and substance P. Plasma cortisol and substance P values in band and surgically castrated animals are provided in Table 2 and Fig. 1 The plasma cortisol values were significantly elevated in the control animals compared to the treated animals (*P <* 0.05) on day 0 and 1 for band castrated animals and day 0 for surgical castrated animals. The plasma substance P concentrations were significantly elevated in the control animals compared to the treated animals (*P <* 0.05) on day 0 and day 1 for both the band castrated animals and surgical castrated animals.

**Table 2:** Plasma Cortisol and Substance P values in Band and Surgically Castrated Animals

Day of Study	Treatment	Plasma Cortisol (ηmol/L)				Substance P (pg/L)			
		Band Castrated		Surgical Castrated		Band Castrated		Surgical Castrated	
		Mean (SE)	*P* value	Mean (SE)	*P* value	Mean (SE)	*P* value	Mean (SE)	*P* value
Day -1	Meloxicam	14.2 (1.6)	0.8519	16.4 (2.3)	0.6041	243.9 (16.4)	0.4679	249.8 (7.8)	0.4849
	Control (Saline)	13.2 (1.4)		18.7 (1.9)		268.2 (15.6)		244.5 (11.5)	
Day 0	Meloxicam	23.9 (1.2)	0.0032	39.6 (5.5)	0.0421	243.7 (13.9)	0.0012	267.9 (11.2)	0.0137
	Control (Saline)	36.1 (4.5)		58.0 (7.7)		340.5 (23.0)		314.7 (13.4)	
Day 1	Meloxicam	29.6 (5.3)	0.0340	14.1 (2.1)	0.8785	261.8 (15.5)	0.0181	260.6 (8.1)	0.0424
	Control (Saline)	38.4 (3.6)		18.2 (5.1)		335.5 (23.8)		304.4 (17.0)	
Day 2	Meloxicam	33.4 (5.4)	0.8846	9.3 (1.7)	0.0808	251.6 (14.9)	0.0680	273.2 (10.4)	0.3947
	Control (Saline)	33.6 (4.3)		13.7 (2.4)		295.2 (16.3)		301.1 (17.0)	
Day 3	Meloxicam	27.6 (6.7)	0.6783	14.9 (2.0)	0.4581	253.7 (12.7)	0.5338	333.7 (11.2)	0.8103
	Control (Saline)	28.7 (6.4)		19.6 (3.0)		264.9 (16.4)		334.8 (5.3)

### Accelerometer behavior data

**Fig. 2:**
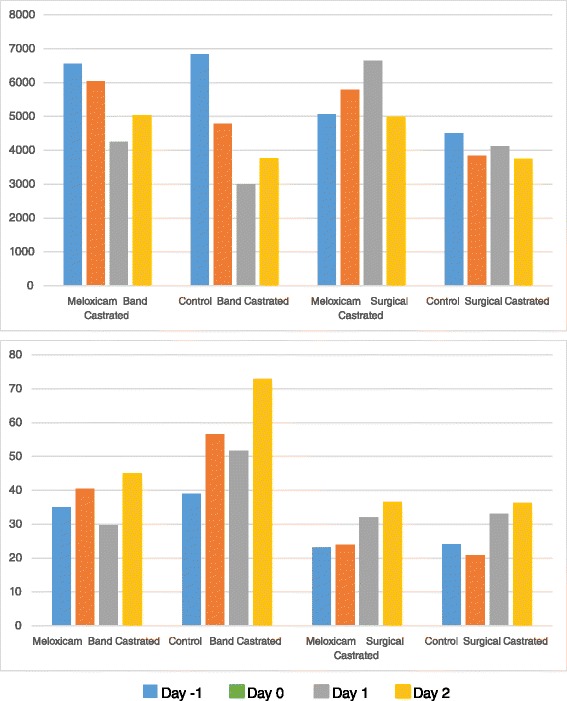
Motion Index (Upper) and Number of Lying Bouts (Lower) in band and surgically castrated calves on day -1, 0, 1 and 2

Ice Tag data was downloaded from the accelerometers and only times that animals were in their home pen without any external disturbances were included for analysis. For Band Castrated calves (Study 1) analysis periods were: a) Day -1 (3:00 pm) to Day 0 (8:00 am), b) Day 0 (1:00 pm) to Day 1 (10:00 am), c) Day 1 (1:30 pm) to Day 2 (8:30 am); Day 2 (11:30 am) to Day 3 (9:00 am). For Surgical Castrated calves (Study 2) analysis periods were: a) Day -1 (1:30 pm) to Day 0 (8:00 am), b) Day 0 (2:15 pm) to Day 1 (9:00 am), c) Day 1 (10:30 am) to Day 2 (8:30 am); Day 2 (10:00 am) to Day 3 (9:00 am). The behavior data is provided in Table 3 and Fig. 2.

**Table 3:** Accelerometer Behavioral Data in Band and Surgically Castrated Animals

Study Day	Variable	Treatment	Band Castrated			Surgical Castrated		
			Mean	SE	*P* Value	Mean	SE	*P* Value
Day -1 To Day 0 (pre-castration)	Motion Index	Meloxicam	6563	431.2	0.6783	5066	236.6	0.0771
		Control	6835	391.9		4510	200.2	
	% Time Lying	Meloxicam	62.2	1.1	0.6482	68.2	0.88	0.9131
		Control	61.4	1.6		67.9	0.95	
	Number of Steps	Meloxicam	1728	72.5	0.0537	1397	91.8	0.3051
		Control	2006	101.7		1197	54.3	
	Lying Bouts	Meloxicam	35	3.3	0.4303	23.21	1.5	0.5698
		Control	39	3.9		24.07	1.6	
Day 0 To Day 1	Motion Index	Meloxicam	6041	311.6	0.0144	5786	369.9	0.0001
		Control	4778	320.3		3836	218.8	
	% Time Lying	Meloxicam	62.5	1.1	0.0079	59.7	1.9	0.4450
		Control	68.1	1.5		60.4	1.2	
	Number of Steps	Meloxicam	1630	66.1	0.0340	1650	65.0	0.0028
		Control	1321	106.1		1293	68.4	
	Lying Bouts	Meloxicam	40.5	3.1	0.0095	24.0	1.6	0.1967
		Control	56.5	4.2		20.9	1.5	
Day 1 to Day 2	Motion Index	Meloxicam	4255	216.0	0.0003	6639	910.9	0.0011
		Control	3001	189.2		4114	212.6	
	% Time Lying	Meloxicam	62.4	1.4	0.4068	61.4	0.86	0.0154
		Control	64.5	1.4		58.9	0.66	
	Number of Steps	Meloxicam	1229	59.9	0.0055	1778	91.8	0.0424
		Control	953	75.5		1497	76.5	
	Lying Bouts	Meloxicam	29.7	3.2	0.0045	32.1	3.88	0.6940
		Control	51.7	5.3		33.07	2.53	
Day 2 To Day 3	Motion Index	Meloxicam	5030	379.2	0.0465	4985	382.9	0.0043
		Control	3769	314.4		3748	174.2	
	% Time Lying	Meloxicam	60.7	1.3	0.0815	61.34	1.0	0.6468
		Control	63.9	1.4		60.4	1.0	
	Number of Steps	Meloxicam	1639	71.9	0.0238	1666	81.6	0.0121
		Control	1351	83.0		1429	66.1	
	Lying Bouts	Meloxicam	45.1	8.9	0.0361	36.7	3.7	0.9652
		Control	72.9	12.6		36.3	2.7

For band castrated calves, the motion index was significantly elevated in meloxicam treated animals compared to the control animals (*P <* 0.05) on day 0–1, day 1–2 and day 2–3. The percent of time lying was significantly lower in meloxicam treated animals compared to the control animals (*P <* 0.05) on day 0–1. The number of steps taken was significantly greater in meloxicam treated animals compared to the saline treated animals (*P <* 0.05) on day 0–1, day 1–2 and day 2–3. Band castration control animals had significantly more lying bouts than meloxicam treated animals (*P <* 0.05) on day 0–1, day 1–2 and day 2–3.

In surgically castrated animals the motion index was significantly elevated in meloxicam treated animals compared to the treated animals (*P <* 0.05) on day 0–1, day 1–2 and day 2–3. The percent of time lying was significantly greater in meloxicam treated animals compared to the saline treated animals (*P <* 0.05) on day 1–2 for surgically castrated animals. The number of steps taken was significantly elevated in meloxicam treated animals compared to the saline treated animals (*P <* 0.05) on day 0–1, day 1–2 and day 2–3. There was no difference in the number of lying bouts between control animals and meloxicam treated animals (*P >* 0.05).

### Visual analog scale (VAS)

**Fig. 3:**
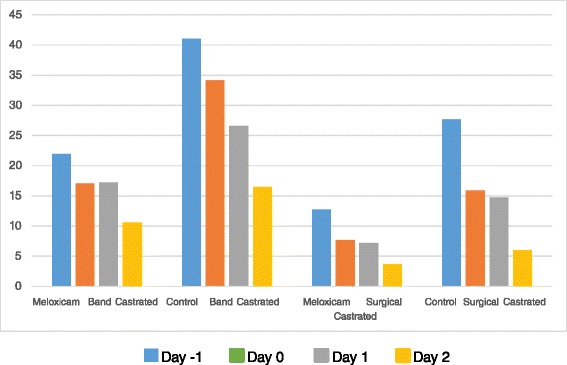
Visual Analog Score (VAS) of in band and surgically castrated calves on day -1, 0, 1 and 2

The mean total Visual Analog Scale (VAS) score the three experienced observers used to identify painful behaviors in cattle are provided in Table 4 and Fig. 3. The data is expressed as a percentage of the total line with 0 % assigned to animals with no observed pain associated behaviors and 100 % assigned to animals with severe pain behaviors (e.g. kicking, stretching, changing of position, arched back, standing alone in the pen). The VAS scores are higher in all band castrated animals compared to all surgical castrated animals. Band castrated animals receiving Meloxicam Oral Suspension demonstrated significantly less painful behaviors (*P <* 0.05) than saline treated controls on observation day 0, day 1 and day 2. Surgical castrated animals receiving Meloxicam Oral Suspension demonstrated significantly less painful behaviors (*P <* 0.05) than saline treated controls on observation for day 0, day 1 and day 2. The VAS was compared between band and surgical castrated calves. For both meloxicam and control castrated calves there was a significantly higher scores on days 0, 1 and 2 in Band castrated calves compared to surgical castration.

**Table 4:** Mean Visual Analog Scale scores Behavioral Data in Band and Surgically Castrated Animals

Study Day	Band vs Surgical Castration *P* value		Treatment	Band Castrated			Surgically Castrated		
				Mean (%)	Standard Error	*P* Value	Mean (%)	Standard Error	*P* Value
Day -1	Control	Meloxicam	Meloxicam	0	0	1.000	0	0	1.000
			Control (Saline)	0	0		0	0	
Day 0	0.0001	<0.0001	Meloxicam	21.97	3.10	0.0001	12.70	2.12	0.0001
			Control (Saline)	41.07	3.73		27.64	3.04	
Day 1	0.002	0.0002	Meloxicam	17.05	2.66	0.0001	7.67	1.53	0.002
			Control (Saline)	34.17	3.28		15.92	2.25	
Day 2	0.0136	0.0031	Meloxicam	17.25	2.88	0.0056	7.20	1.51	0.0136
			Control (Saline)	26.61	3.29		14.69	2.39	
Day 3	0.0515	0.1296	Meloxicam	10.61	1.78	0.0515	3.73	0.72	0.1296
			Control (Saline)	16.44	2.37		6.04	0.98

### Tail pedometers

Tail pedometer data was collected on the belief that pain associated with castration in calves would result in more tail swishes. Pedometers were easily attached to the tail base and tail swishes could be recorded. Animals rarely lost their pedometers and they were replaced the next day during processing. Data from animals that lost their pedometers was not used in the analysis. Pedometer data is provided in Table 5. There was no difference in the number of “steps” recorded on the tail pedometers between control animals and meloxicam treated animals (*P >* 0.05) following band and surgical castration. The “steps” on the pedometers represent the number of tail swishes.

**Table 5:** Mean Pedometer Recordings (Tail Swishes) in Band and Surgically Castrated Animals

Study Day	Treatment	Band Castration			Surgical Castration		
		Mean (swishes)	Standard Error	*P* Value	Mean (swishes)	Standard Error	*P* Value
Day –1 to 0	Meloxicam	5425	452	0.4068	16095	2567	0.0275
	Control (Saline)	7041	1094		9011	1297	
Day 0 to 1	Meloxicam	8575	1327	0.1161	19168	2414	0.058
	Control (Saline)	8135	1350		12934	1775	
Day 1 to 2	Meloxicam	7730	1402	0.6625	20674	3683	0.7107
	Control (Saline)	11961	2238		17559	2269	
Day 2 to 3	Meloxicam	12819	2172	0.1264	20394	4137	0.9085
	Control (Saline)	17865	2791		16945	2595	

### Inflammation scoring and measurement

Generally, swelling in the band castrated calves was minimal proximal to the band in the inguinal area. The scrotal inflammation score was significantly less in the meloxicam treated animals compared to the control animals (*P <* 0.05) for band castrated animals on day 2 and 3 (Table 6). In surgical castrated animals the scrotal tissue swelling could be easily objectively evaluated using digital calipers (Table 6). The scrotal inflammation (based on scrotal swelling) was significantly less in the meloxicam treated animals compared to the control animals (*P <* 0.05) for surgical castrated animals on day 1, day 2 and 3. There was no evidence of infections in any animal in the study.

**Table 6:** Mean Swelling Scores (Band Castrated Animals) and Mean Mid-Scrotal Diameter (Surgically Castrated Animals)

Study Day	Treatment	Band Castration			Surgical Castration		
		Mean Score	Standard Error	*P* Value	Mean (mm)	Standard Error	*P* Value
Day 0	Meloxicam	0	0	1.000	10.97	0.27	0.4845
	Control (Saline)	0	0		11.28	0.28	
Day 1	Meloxicam	0.067	0.067	0.1577	26.53	1.21	0.0001
	Control (Saline)	0.267	0.118		41.01	2.69	
Day 2	Meloxicam	0.200	0.107	0.0297	33.54	2.54	0.0154
	Control (Saline)	0.600	0.131		43.58	2.85	
Day 3	Meloxicam	0.467	0.165	0.0232	37.81	2.26	0.0246
	Control (Saline)	1.067	0.182		48.82	3.10	

## Discussion

Cattle are able to mask pain and stress as an important component to survival. The development of appropriate cattle pain model systems and robust markers are essential for the demonstration of pain and pharmacological relief of pain [3, 4]. The study protocol was designed to focus on robust indicators that have been established and validated previously [3, 4]. The investigators have also learned that the breed and age of animals also play a critical role in demonstration of pain and relief of pain. Beef breeds that are over 250 kg have difficulty in displaying demonstrations of pain [3, 4], but this does not mean that they do not feel pain. Well acclimated dairy calves which are 100 to 300 kg body weight appear to respond best to painful procedures such as castration and dehorning as well as interventions that reduce or eliminate the pain [3, 4]. For this reason we have selected dairy bull calves for this study. In this study the calves were brought to the study site at a young age and were handled daily and frequently by farm employees. These animals were ideal subjects as they were not stressed by handling and therefore the physiological and behavioral responses to the meloxicam could be readily measured [3, 4].

Compounded oral meloxicam preparations have been used previously in North America for control of pain and inflammation for dehorning and castration [3, 4]. Meloxicam Oral Suspension is the first commercial oral meloxicam available for cattle in North America. This formulation has proven to be stable, effective and safe for cattle as part of the regulatory submission. The long duration of activity (3 days) and versatility of oral delivery provides the veterinarian and producer a product that can address their animal welfare needs.

It has been demonstrated that heart rate increases over baseline levels by stressful events such as castration, dehorning, and branding [3, 12, 13]. The duration of the increased heart rate over baseline has been reported to last for several hours after the painful stimulation. Recently meloxicam was shown to significantly reduce heart rates in scoop dehorned cattle which was attributed to reduction in pain and stress associated with NSAID treatment [14]. In this study, there were no uncastrated control animals but the heart rate was significantly increased in control animals over meloxicam treated animals for 10 h after the castration procedure. This effect was observed in both band castrated and surgically castrated animals.

There are many studies that have shown an increase in plasma cortisol associated with castration in cattle [3, 15, 16]. The peak cortisol concentration is reported to occur within 30 min after castration [3]. Dairy calves also appear to be more responsive than beef bulls and it is believed that beef calves have a higher tolerance to pain [3]. Plasma cortisol has been shown to be significantly reduced in castrated calves receiving NSAIDs before castration [16]. In this study plasma cortisol was significantly reduced on day 0 and day 1 which indicates that animals receiving meloxicam are in less pain and are under less stress than control calves. As expected, the difference was most pronounced on day 0 (approximately 4 h after castration).

Substance P is an 11-amino acid neuropeptide that regulates the excitability of dorsal horn nociceptive neurons and is present in areas of neuroaxis involved in pain, stress and anxiety [3]. It has been shown to be elevated with soft tissue injury, castration and dehorning [3, 14, 17]. In this study, plasma substance P was elevated in control animals over meloxicam treated animals on day 0 (approximately 4 h after castration) and day 1 (approximately 20 h after castration). This is similar with that observed previously with dehorned dairy bulls [14].

There are several manufacturers of accelerometers that can be attached to the leg of cattle and provide long term, unbiased and validated behavioral data. The ICE tags used it this study have been validated and extensively used in cattle for behavioral assessment of pen designs and pharmaceutical intervention (www.icerobotics.com). The accelerometers allow objective monitoring of animal behavioral changes as animals in pain have certain behavioral characteristics: 1) they spend more time lying, 2) they stand up and lay down more frequently (uncomfortable), 3) they walk less (fewer steps). The ICE tag accelerometers are able to record these events as well as generate a movement index. The higher the movement index, the more the animal is moving throughout the pen. In this study meloxicam had a significant effect on several behavioral parameters: In both band and surgical castrated animals the motion index and the number of steps were significantly greater in meloxicam treated animals on day 0 (first 24 h after castration), day 1 (24 to 48 h after castration) and day 2 (48 to 72 h after castration). This suggests that animals receiving meloxicam were in less pain and there was less swelling allowing the animals to move more freely throughout the pen. There were a greater number of lying bouts in control animals over meloxicam treated animals in only the band castrated animal but this was observed on days 0, 1 and 2. This suggests that band castration makes it more uncomfortable for the animals to remain in the lying position for extended periods of time. The percent of time lying was only significantly more in control animals on day 0 (band castrated) and day 1 (surgical castrated). These results are in agreement with observations previously published where meloxicam demonstrated evidence of changes in calf behavior associated with castration and dehorning [7, 18]. In these studies meloxicam treated animals spent more time walking and less time lying down. These studies concluded that lying behavior and movement are good indicators of animal well-being and pain.

Visual Analog Scale (VAS) score is a subjective method to rate the level of pain in animals. This method permits evaluation of the level of pain by observing behaviors that cannot be easily objectively recorded. These include, stretching, belly kicks, licking, foot stomping and ear position. The VAS scoring was significantly greater (more pain) in control calves over meloxicam treated animals on days 0, 1 and 2 for both band and surgical castrated animals. This supports the previous studies where behavioral evaluation demonstrated that meloxicam had a positive, ameliorative effect on behavioral changes in dehorned animals [7, 18].

Tail swishing is a response to a number of stimuli including 1) flies, 2) skin irritation and 3) local painful stimuli. Castrated animals generally swish their tails more frequently due to localized pain. It was hoped that tail pedometers would be able to identify positive behavioral effects of meloxicam by reducing tail swishing. In this study there was considerable variation among animals with respect the number of “steps” recorded on the tail pedometers. There was no difference between the number of steps in control and meloxicam treated animals. This method may not be sensitive enough to demonstrate a difference and/or the number of flies that stimulated tail swishing masked any swishing due to painful stimuli.

Meloxicam has been shown to be an effective anti-inflammatory agent in many veterinary species including cattle [19, 20]. This study permitted the subjective and objective evaluation of inflammation at the band and surgical wound site following castration. There was a pronounced effect of meloxicam in surgical castrated animals and the effect was less in band castrated animals. We have recently shown that oral meloxicam has a significant effect in reduction of swelling in castrated horses [21].

## Conclusion

Meloxicam Oral Suspension was able to significantly reduce pain response behaviors and physiological responses to pain in band castrated and surgical castrated calves for up to 72 h following a single oral treatment of 1 mg/kg body weight. Meloxicam Oral Suspension was also able to significantly reduce inflammation in band castrated and surgical castrated calves. Now that oral meloxicam is available to Canadian Veterinarians and producers additional benefits to cattle and other food animal species may be recognized. The availability of this product should improve the welfare of cattle and other larger companion and food animal species.

## Abbreviations

NSAID, nonsteroidal anti-inflammatory drug; VAS, visual analog score
